# Characterization of poly(A) and poly(T) tail lengths in plasmid DNA by liquid chromatography high-resolution mass spectrometry

**DOI:** 10.1007/s00216-024-05654-6

**Published:** 2024-11-16

**Authors:** Nour Al Turihi, Delphine Allouche, Maëlle Quéré, Mathieu Scuiller, Isabelle Legastelois

**Affiliations:** RNA Sciences, Sanofi, 1541 av. Marcel Mérieux, 69280 Marcy l’Étoile, France

**Keywords:** Mass spectrometry, Poly(A) tail length, MRNA, DNA template

## Abstract

Rapid advances in messenger RNA (mRNA) technology necessitate effective analytical methods. This study describes the development of a novel *in vitro* method using ion-pair reversed-phase liquid chromatography coupled to high-resolution mass spectrometry (IP-RP-LC-HRMS) for assessing the poly(A) and complementary poly(T) tail lengths directly into the DNA template used to manufacture mRNA. Briefly, after the validation of poly(A) tail length in the plasmid by Sanger sequencing, double-stranded DNA fragments containing these tails in the plasmid of interest were amplified by the polymerase chain reaction (PCR), purified on silica column, and digested with restriction enzymes ClaI and HindIII. Gel or capillary electrophoresis confirmed sample quality and enzymatic digestion efficiency. Subsequently, poly(A) and complementary poly(T) tails were extracted and analyzed by LC-MS to determine their length and heterogeneity at a single-nucleotide resolution. Three DNA templates containing poly(A) tail lengths of 60A-G, 95A, or 108A were studied. LC-MS results correlated well with Sanger sequencing, identifying major populations of 60A-G, 95A, or 108A. Surprisingly, unlike Sanger sequencing, LC-MS analysis revealed minor poly(A) populations with lengths longer or shorter than the theoretically encoded poly(A) tail length. This finding could be explained by (i) the slippage of bacterial DNA polymerase I during plasmid replication in bacterial culture, which occurs on repeat mononucleotide sequences, or (ii) the slippage of Q5® High-Fidelity DNA Polymerase during PCR amplification. In conclusion, the method is easy, rapid, and accurate and could replace Sanger sequencing to assess the poly(A) and complementary poly(T) tail lengths in plasmid DNA.

**Supplementary Information:**

The online version contains supplementary material available at 10.1007/s00216-024-05654-6.

## Introduction

The COVID-19 pandemic popularized the use of messenger RNA (mRNA) in a range of prophylactic and therapeutic approaches. Indeed, this technology holds enormous potential to help prevent or combat infectious and incurable or serious diseases, such as cancer, autoimmune, and certain rare diseases [1]. Unlike traditional virus-based and protein-based vaccines, mRNA vaccines offer greater benefits due to their faster design and production as well as their flexibility and cost-effectiveness [2, 3].

Conventional mRNA vaccines are usually composed of lipid nanoparticles (LNPs), which encapsulate mRNA molecules. LNPs protect mRNA from degradation and act as drug-delivery vehicles by carrying mRNA into the cells [4]. Once inside the host cells, mRNA uses the cell translational machinery to produce the antigen of interest and initiate an adaptive immune response [5]. The mRNA strand is produced by an in vitro transcription (IVT) reaction from a DNA template. The mRNA structure is composed of several components, including the cap structure at the 5′ end, the 5′ untranslated region (UTR), the coding sequence (CDS), the 3′ UTR region, and, finally, the polyadenosine [poly(A)] tail at the 3′ end [6]. The poly(A) tail is essential for the translation and stability of mRNA [7, 8]. Two primary strategies exist for adding a poly(A) tail to the IVT-mRNA structure: encoding it directly into the DNA template or enzymatically adding it post-IVT [9]. Both approaches work but have their own drawbacks. Post-IVT addition results in more heterogeneous tail lengths compared to the plasmid-encoded poly(A) tail [10]. Conversely, amplifying plasmids with long poly(A) tails in *Escherichia coli* leads to plasmid instability and tail reduction during culture passages. One way to avoid this plasmid instability is to incorporate non-poly(A) spacers inside the poly(A) DNA sequence [11, 12].

When using the IVT-mRNA approach, characterizing the poly(A) tail length heterogeneity is crucial as mRNA is used for therapeutic or vaccine aims [13]. Many regulatory texts such as the *United States Pharmacopeia* defined the poly(A) tail as a critical quality attribute for mRNA-based therapeutics and vaccines.

Numerous analysis methods can be used to characterize the poly(A) tail heterogeneity present in a single-stranded RNA [9, 10, 14–17]. These methods typically involve either RNase T1 cleavage, which specifically targets the 3′-end of guanosine in single-strand RNA, or DNA probe hybridization. In the latter method, a specific DNA probe hybridizes to the 3′ end of mRNA before the poly(A) tail, allowing site-specific cleavage by RNase H to release the poly(A) tail. Once extracted, the poly(A) tail can be characterized using several analytical methods, such as gel electrophoresis or ion-pair reversed-phase liquid chromatography coupled to ultraviolet (IP-RP-LC-UV). However, these methods lack sufficient resolution to assess the poly(A) tail heterogeneity [15]. Beverly et al. showed that IP-RP-LC coupled to high-resolution mass spectrometry (IP-RP-LC-HRMS) could be used to characterize the mRNA poly(A) tail length distribution with a satisfactory resolution (to the nearest nucleotide) [10]. Recently, Di Grandi et al. demonstrated that capillary gel electrophoresis could also have a sufficient resolution to assess the poly(A) tail length distribution on mRNA [17]. Nonetheless, these two methods have been developed to assess the poly(A) tail on mRNA exclusively. Different sequencing technology platform methods such as Sanger or next-gene sequencing are required to assess the length of poly(A) tail on DNA [18]. Although these methods allow for reading the exact composition of a nucleotide sequence — and not just determining the sequence length — these techniques may lack in precision. Indeed, detecting multiple populations is complex and the DNA amplification step can be a source of error, particularly for poly(A) tails. Moreover, these sequencing methods often struggle to accurately determine the correct number of adenines [19].

In order to avoid the shortcomings of the aforementioned approaches, we developed a unique workflow comprising an IP-RP-LC-HRMS method for assessing the poly(A) tail length heterogeneity directly into the plasmid DNA and at a single-nucleotide resolution. This analytical method is orthogonal to existing methods and is designed to be quick and easy to implement. Overall, it is similar to the one developed by Sattler et al. to analyze short synthetic DNA oligonucleotide (100 nT) [20]. Summarily, this approach requires amplifying the DNA fragments containing the poly(A) tail by polymerase chain reaction (PCR), purifying the result on a silica column, and then digesting the collected fraction with the ClaI and HindIII enzymes that cleave DNA at the restriction sites bordering the poly(A) tail. Afterward, the poly(A) tail, along with its complementary poly(T) tail, is analyzed by IP-RP-LC-HRMS for determining its length and heterogeneity at a single-nucleotide resolution. This study helped assess the length of poly(A) tails and complementary poly(T) tail lengths of three DNA templates containing, in theory, poly(A) tails of 60A followed by a G (60A-G), 95A, or 108A using IP-RP-LC-HRMS and compare the results with those obtained by Sanger sequencing, agarose and acrylamide gels, and finally capillary electrophoresis.

## Materials and methods

### Plasmid constructs

**Fig. 1:**
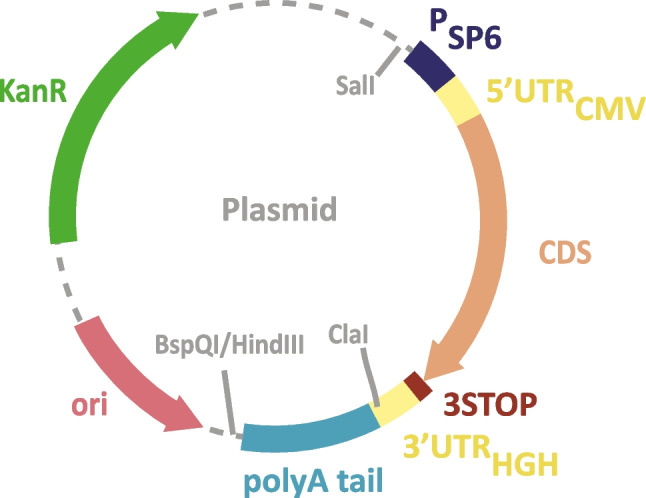
Schematic plasmid map. The three plasmids were built from the same backbone composed of a pUC-type origin of replication (*ori*), a kanamycin-resistance (KanR) cassette, and an IVT cassette. The two variable elements of this IVT cassette were the CDS [HA(H3) or eGFP] and the poly(A) tail (60A+G or 95A or 108A)

The three plasmids used presented a pUC-type *ori* sequence, a kanamycin-resistance cassette (*aph(3*′*)* gene) and an “IVT” cassette flanked by restriction sites SalI and HindIII (Fig. 1). The IVT cassette contained an SP6 promoter and a coding sequence flanked by 5′ and 3′UTR, with the poly(A) at the end of the 3′UTR. The plasmids named “HA(H3)_60A-G” had a CDS encoding for hemagglutinin subtype 3 [HA(H3)] from the A/Singapore/INFIMH-16-0019/2016(H3N2) influenza strain and 60A followed by a G after the 3′UTR. The plasmid named “HA(H3)_95A” contained the same CDS with a pure poly(A) of 95A, while the plasmid named “GFP_108A” encoded for the enhanced green fluorescence protein (eGFP) contained a pure poly(A) of 108A (Supplementary Table 1).

Plasmids were constructed by Gibson assembly of synthetic DNA fragments with 25-bp homologous ends using the NEBuilder® HiFi DNA Assembly Master Mix (reference: E2621, New England Biolabs). After a heat-shock transformation in NEB® Stable Competent *E. coli* strain (reference: C3040, New England Biolabs), isolated clones were selected from an overnight culture on an LB agar plate (reference: L7025, Sigma-Aldrich), containing 50 µg/mL kanamycin (reference: K0254, Sigma-Aldrich). Plasmid sequences were validated from miniprep cultures by Sanger sequencing and then amplified in a shake-flask at 30 °C with shaking (at 220 rpm) in an LB medium (reference: 10855021, Gibco) for 20 h, followed by a purification using QIAfilter Maxiprep Plasmid Kit (reference: 12263, Qiagen) or NucleoBond Xtra Midi kit (reference: 740410.50, Macherey Nagel).

### Poly(A) tail region sequence validation by Sanger sequencing

**Fig. 2:**
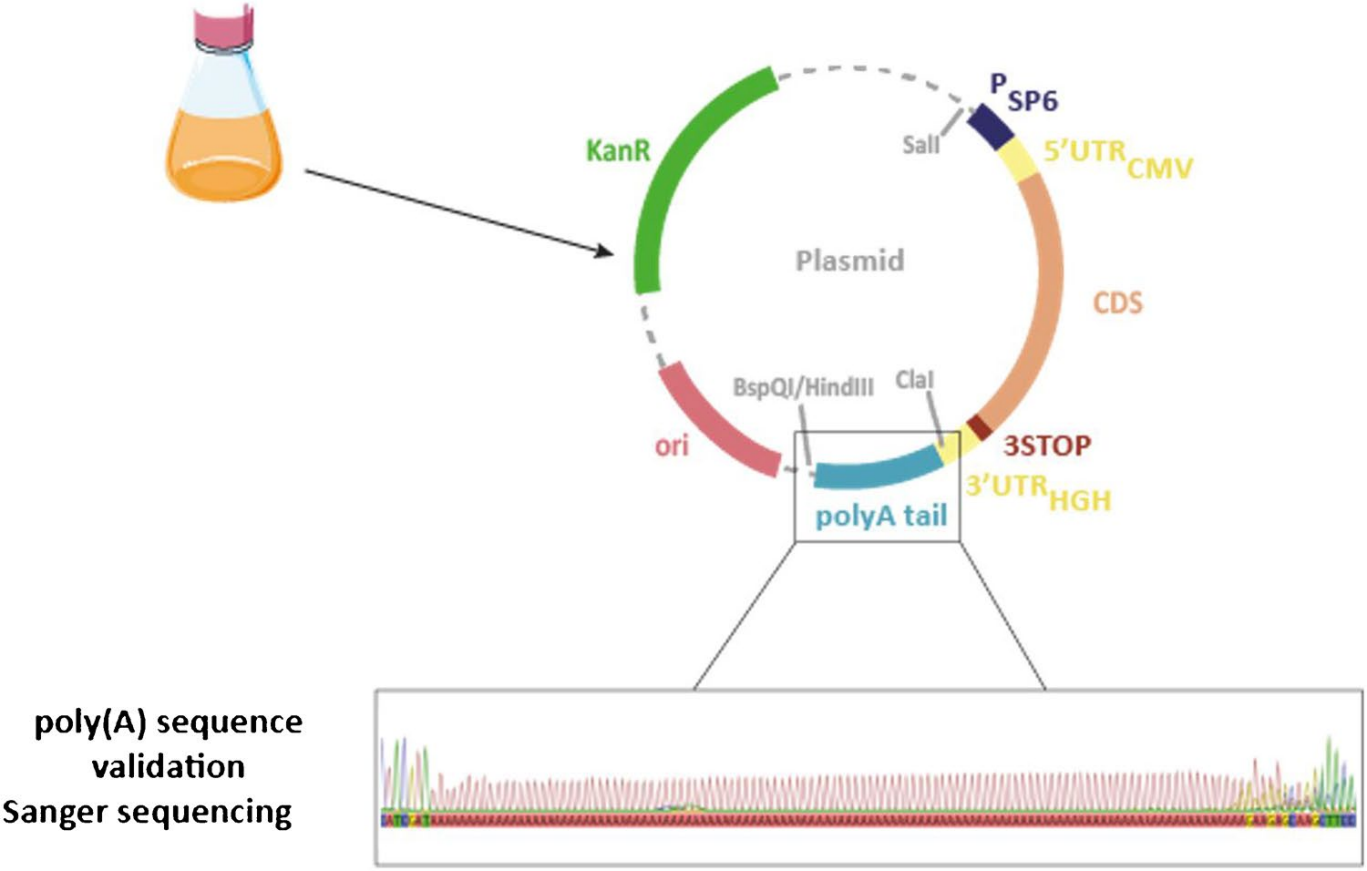
A poly(A) tail sequence obtained after DNA amplification of the poly(A) region. The length of the poly(A) tail is validated at first by Sanger sequencing after amplification using specific primers

The poly(A) tail region in different plasmids was amplified using the BigDye™ Terminator v3.1 Cycle Sequencing Kit (reference: 4376486, Applied Biosystems) according to the supplier protocol by mixing the plasmid and specific primers (forward upstream primer: TAATAGTGACGGGTGGCATC or reverse downstream primer: TTACCGCCTTTGAGTGAGC) with the RR100 reagent and the buffer from the kit. Incubation was performed in a thermocycler (Verity 96 well Therma cycler, Applied Biosystems) according to the supplier protocol. The samples were then purified using BigDye™ X Terminator Purification kit (reference: 4376486, Applied Biosystems) in accordance with the supplier’s recommendations. Purified samples were sequenced on the SeqStudio Flex Genetic Analyzer from Thermo Fisher Scientific (Fig. 2).

### DNA amplification and enzymatic digestion of samples for HPLC/MS and electrophoresis analysis

**Fig. 3:**
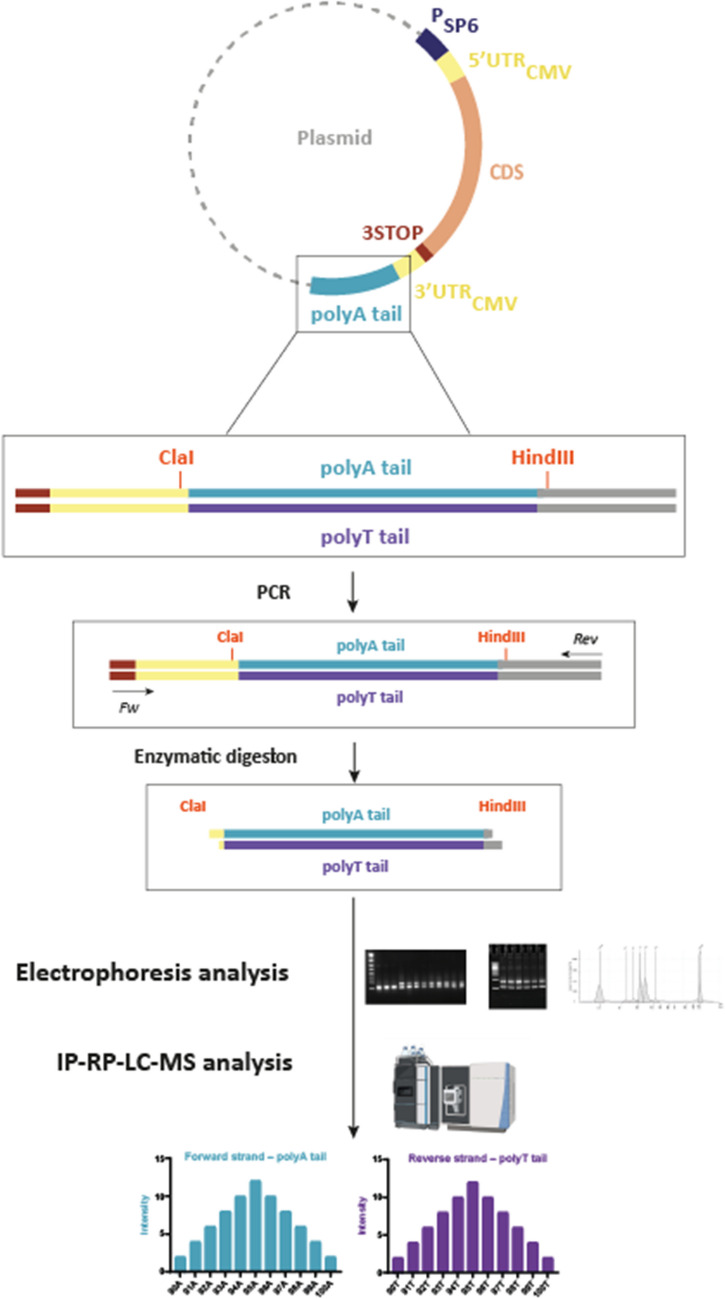
Sample preparation for determining poly(A) tail lengths in plasmid DNA. DNA fragments containing the poly(A) tail were amplified by PCR, purified on silica column, and digested with restriction enzymes ClaI and HindIII. Then, the digested fragments were analyzed using either the selected electrophoresis techniques or LC-MS for determining tail length and heterogeneity at a single-nucleotide resolution

The poly(A) tail region was amplified by PCR from 5 ng of the different plasmids by using the Q5 Master Mix (reference: M0494L, New England Biolabs) with 500 nM of specific primers (Fw: 5′-TAATAGTGACGGGTGGCATC-3′ and Rev: 5′-GAGCGCAACGCAATTAATGT-3′) (Fig. 3). After silica column purification according to the manufacturer’s recommendations (PCR and Gel Clean Up, Macherey Nagel; reference: 740609.50), the amplicon concentration was measured by a NanoDrop™. The purified amplicons were digested by two restriction enzymes ClaI and Hind III (Thermo Fisher Scientific) using 1 U or 1 μL of enzyme for 1 μg of DNA in the reaction mix, with incubation at 37 °C for 15 min. A second round of silica column purification was performed, and the amplicon concentration was measured again by NanoDrop™. The concentration of the purified double-stranded DNA (dsDNA) fragment was approximatively 150 ng/μL.

### Electrophoresis analysis

The DNA fragments were loaded on agarose gel, at a rate of 150 ng per well on 2% of agarose gel (UltraPure™ Agarose, reference: 16500, Invitrogen) and 75 ng per well on 10% of Tris Borate EDTA (TBE) acrylamide gel (Life Technology). For capillary electrophoresis (TapeStation analysis), a concentration of 25 ng/µL was analyzed on D1000 ScreenTape chip (reference: 5067-5582, Agilent Technology) following the instructions from the manufacturer using D1000 reagents (reference: 5067-5583, Agilent Technology). SYBR Gold nucleic acid (reference: S11494, Invitrogen) was used as DNA stain.

### IP-RP-LC-HRMS analysis

The poly(A) tails were analyzed using an IP-RP-LC-HRMS, composed of a Vanquish Flex liquid chromatography (Thermo Fisher Scientific, Bremen, Germany) coupled in-line with an Orbitrap Exploris™ 240 (Thermo Fisher Scientific, Bremen, Germany). DNA fragments were separated on DNAPac™ RP column (C18, 2.1 × 100 mm, 4 µm) using a gradient of solvent A, which composed of H_2_O with 30 mM of hexafluoroisopropanol (HFIP; reference: 147540250, Acros Organics), 10 mM of triethylammonium acetate (TEAA; reference: 90357, Sigma-Aldrich), and 1.2 mM of triethylamine (reference: 90338, Sigma-Aldrich) and solvent B, which composed of EtOH/H_2_O 50/50 (v/v) with 20 mM of TEAA at a flow rate of 0.3 mL/min. The gradient profile for elution started with 0.7 min at 1% solvent B, followed by a 24.3-min linear gradient increasing from 1 to 45% solvent B. Subsequently, there was a 1-min linear gradient from 45 to 80% solvent B. The system held at 80% solvent B for 1.5 min, before returning to 1% solvent B in a 5.5-min linear gradient. The column temperature was set to 65°C and the post-column cooler to 55 °C. The injected volume employed for analysis was 20 µL of the purified dsDNA fragment sample, that is, approximately 3 µg of DNA.

The MS data were acquired on an Orbitrap Exploris™ 240 as follows: All MS1 spectra were acquired over m/z range of 400–2000 with a resolution of 15,000 at m/z 200. The automatic gain control was set to accumulate up to 100%, with a maximum injection time of 100 ms and one microscan. The electrospray ionization source operated in negative polarity mode. The temperatures of the ion-transfer tube and the vaporizer were set at 300°C and 200°C, respectively. The spray voltage was set at 3 kV. The sheath, sweep, and auxiliary gas pressures were set at 50, 1, and 10 arbitrary units, respectively.

### IP-RP-LC-HRMS data analysis

The data were processed using Thermo BiopharmaFinder (v 5.1) using the intact mass analysis workflow. ReSpect and Sliding Windows deconvolution algorithms were used with an offset of 20% and a width of 0.1 min. Other deconvolution parameters were set as follows: output mass ranges were set at 10,000 to 100,000, a deconvolution mass tolerance was 30 ppm, charge state ranges were set at 10 to 100 and 6 to 10 for low and high model masses, relative abundance and quality thresholds were set at 0, peak detection quality measure was set at 95%, peak model width factor was set at 1, and intensity threshold scale was set at 0.01. All DNA theorical sequences were entered manually into BiopharmaFinder for an automatic identification of compound masses. The relative intensities of deconvoluted molecule signals are given for information only, as they depend on numerous factors such as the deconvolution parameters selected and differences in molecule ionization.

## Results and discussions

The poly(A) tail lengths of three DNA templates containing poly(A) tail of 60A-G, 95A, and 108A were analyzed using multiple techniques: Sanger sequencing, agarose and acrylamide gels, capillary electrophoresis, and IP-RP-LC-HRMS.

### Sanger results

**Fig. 4:**
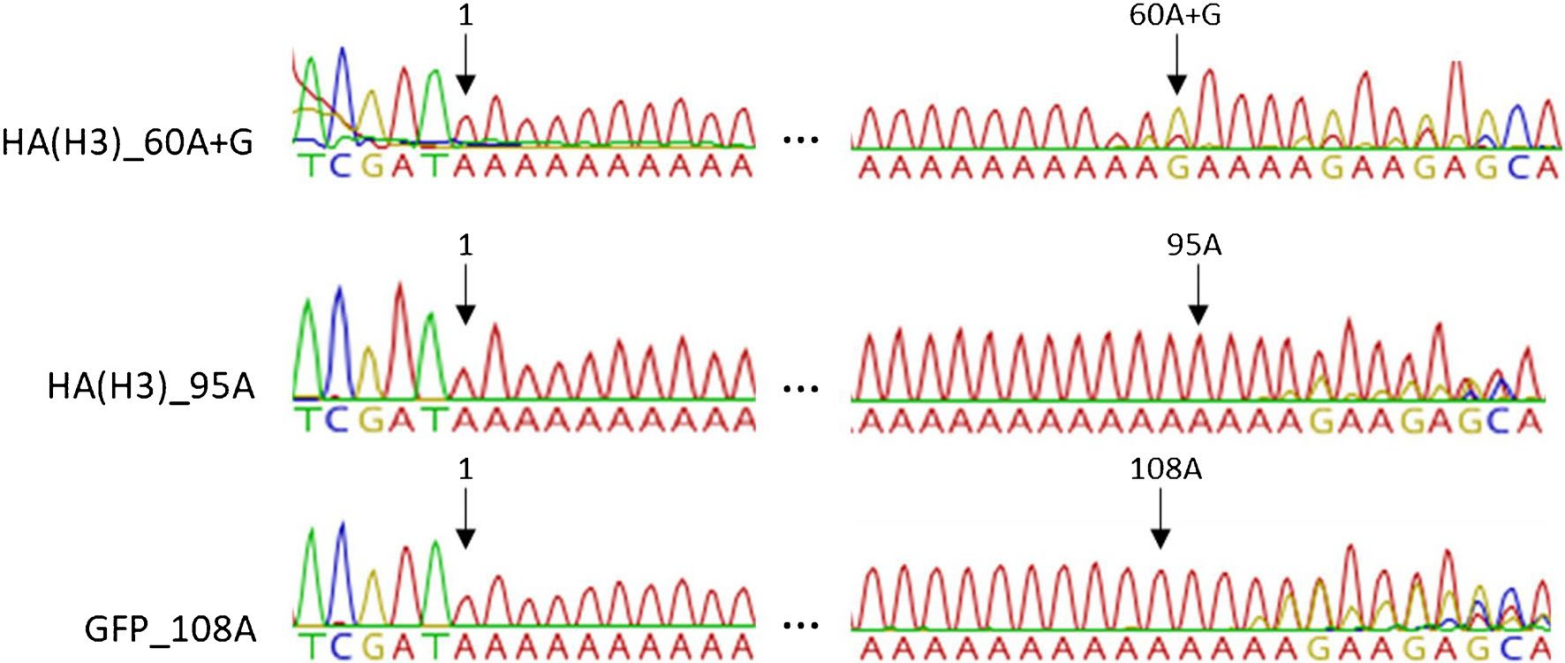
Examples of chromatograms of a poly(A) tail analysis by Sanger sequencing. The three chromatograms are the results of Sanger sequencing of the plasmids with a poly(A) tail of 60A-G, 95A, and 108A respectively. A fully separated signal for each picture on the 5′ side of the poly(A) signal but high background noise on the 3′ side was observed, respectively, on the left and right of the figure

The purified plasmid batches were characterized first by Sanger sequencing after amplifying the poly(A) tail region. The results were interpreted from the chromatograms generated. Nowadays, the Sanger sequencing is the most used technique to obtain the DNA sequence of homopolymeric regions such as poly(A) tails. However, it is well known that the nucleotide composition of CDS and the length of poly(A) can have an impact on a Sanger sequencing analysis. Generally, an analysis of a poly(A) tail longer than 100A is more complex and difficult to have a clean sequence. In our sequencing results, even if the base calling gave a result of 60A-G, 95A, and 108A for the “HA(H3)_60A-G,” “HA(H3)_95A,” and “GFP_108A” plasmids, respectively, a strong inspection of the chromatogram showed second fluorescent peaks in the background (Fig. 4). This background could be due to either the polymerase as explained previously or some minor populations of truncated poly(A) sequence. The issue with Sanger sequencing is that it cannot tell which hypothesis is the right one as the technique only allows to see the majority population but not clearly distinguish several populations on the same chromatogram.

### Electrophoresis analysis

Before an LC-MS analysis, to ensure that the PCR and, more precisely, the digestion had a good efficiency (size and purity), the different digested products coming from “HA(H3)_60A-G,” “HA(H3)_95A,” and “GFP_108A” plasmids were analyzed on agarose and acrylamide gels and by capillary electrophoresis using a TapeStation. Each of these techniques highlighted the presence of fragments of different sizes for each of the three plasmid samples, meaning the purification steps on silica resin were not sufficient to eliminate products from the PCR and digestion such as regions before and after the cleavage sites. For each construction, the different species that might be present in samples are given in Supplementary Table 2 with a total or partial digestion.

**Fig. 5:**
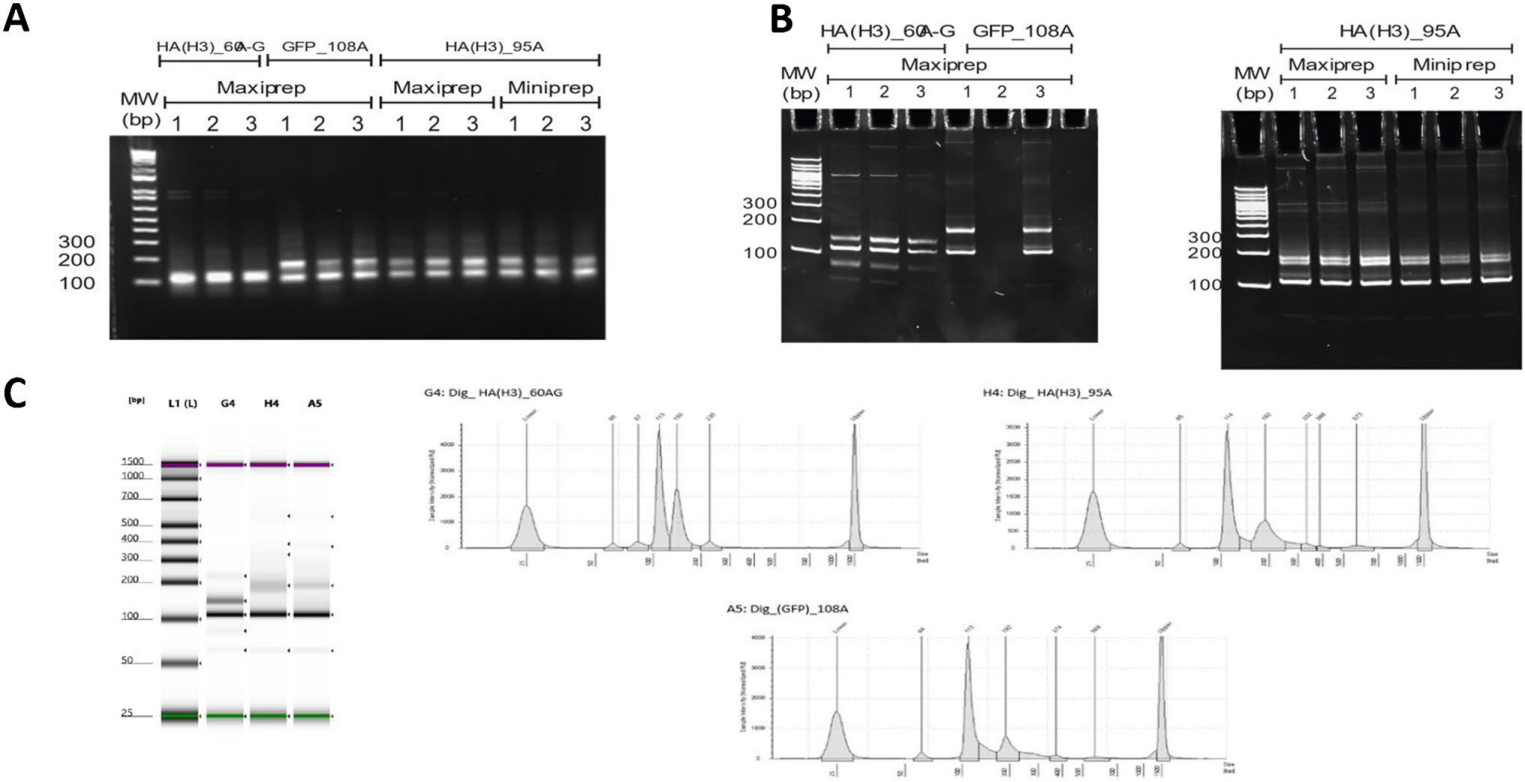
An electrophoresis analysis of amplified and digested poly(A) fragments from the three plasmids. After purification, the digested products were analyzed by different electrophoresis techniques. **A** 2% agarose gel, **B** 10% acrylamide gels, and **C** TapeStation gel and electropherogram (G4 HA(H3)_60A-G; H4: HA(H3)_95A; and A5: GFP_108A). All the samples were analyzed in triplicate (for GFP_108A, a sample was lost during gel loading), except for TapeStation (*n* = 1). MW, molecular weight

The three plasmids presented a major band at around 100 bp, corresponding probably to a partial digestion with ClaI and HindIII in the “HA(H3)_60A-G” plasmid and a total digestion by ClaI and HindIII for the “HA(H3)_95A” and “GFP_108A”plasmids (Fig. 5A). Another band was present at around 200 bp for the “HA(H3)_95A” and “GFP_108A” plasmids, which could correspond to a partial digestion product by ClaI. With the higher resolution 10% acrylamide gel and capillary electrophoresis, the agarose gel 100-bp and 200-bp bands were clearly the sum of different species (Fig. 5B and C). For the “HA(H3)_60A-G” plasmid, two thin bands under 100 bp were detected, corresponding to the size of a total digestion of the plasmid by both restriction enzymes and the downstream cleaved region by HindIII. Then, the upper band around 100 bp corresponded to the partial digestion by ClaI or HindIII. For the “GFP_108A” plasmid, there was an additional band at around 180 bp corresponding to a digestion by ClaI but not by HindIII. Finally, for the “HA(H3)_95A” plasmids, four different populations were observed as included between 100 bp and 200 bp. Among them, a partial digestion with ClaI at 170 bp and a total digestion at 110 bp were found. For TapeStation, the size accuracy is 15% for DNA products of around 15 bp to 300 bp according to the manufacturer’s instructions. Due to these approximative data, we cannot attribute the different bands to a specific species (Supplementary Table 2).

To sum up, the electrophoresis techniques used confirmed that the digestion of the three plasmids was incomplete and that fragments of several sizes were present in the samples. These fragments will be further analyzed by an HPLC/MS. As highlighted previously, using an electrophoresis analysis did not allow to precisely determine the size and the proportion of the different bands, and even less the number of “A” present in the poly(A) tail.

### LC-MS results

**Fig. 6:**
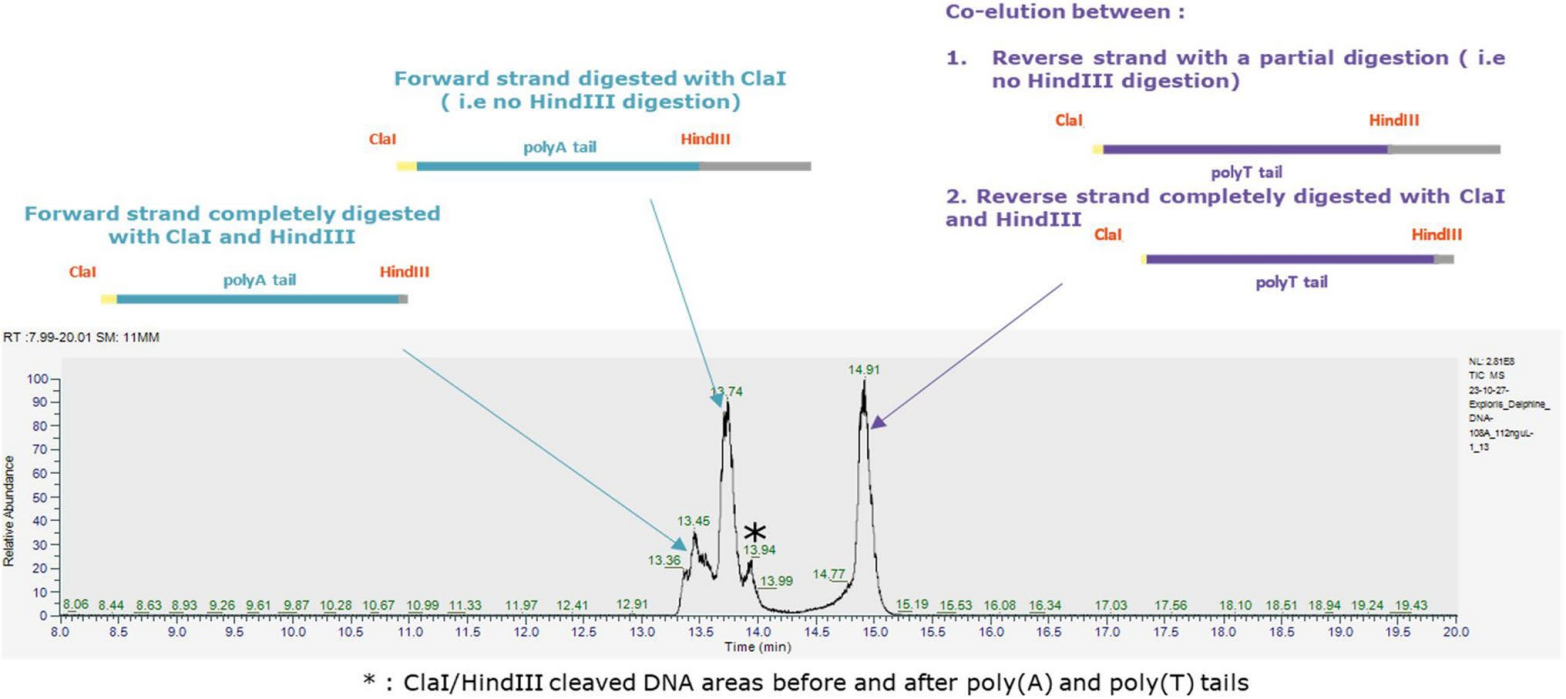
A TIC chromatogram of ClaI/HindIII digested products from plasmid with an encoded poly(A) of 108A. Several peaks were identified that corresponded, after reprocessing data, to ClaI/HindIII undigested, partially, or fully digested products

**Fig. 7:**
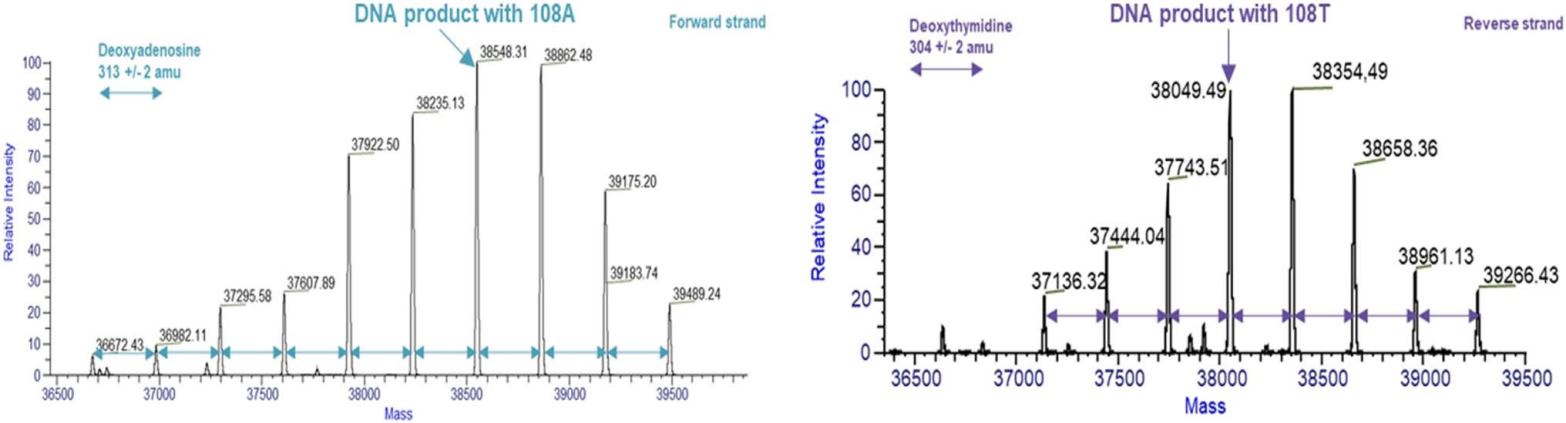
Deconvoluted mass spectrum of ClaI/HindIII fully digested products from plasmid with an encoded poly(A) of 108A. Mass versus spectral intensity is plotted, and the series of peaks separated by the mass of deoxyadenosine (313 ± 2 amu) or deoxythymidine (304 ± 2 amu) represent the different poly(A) and poly(T) tail lengths observed in products from forward and reverse strands, respectively

**Fig. 8:**
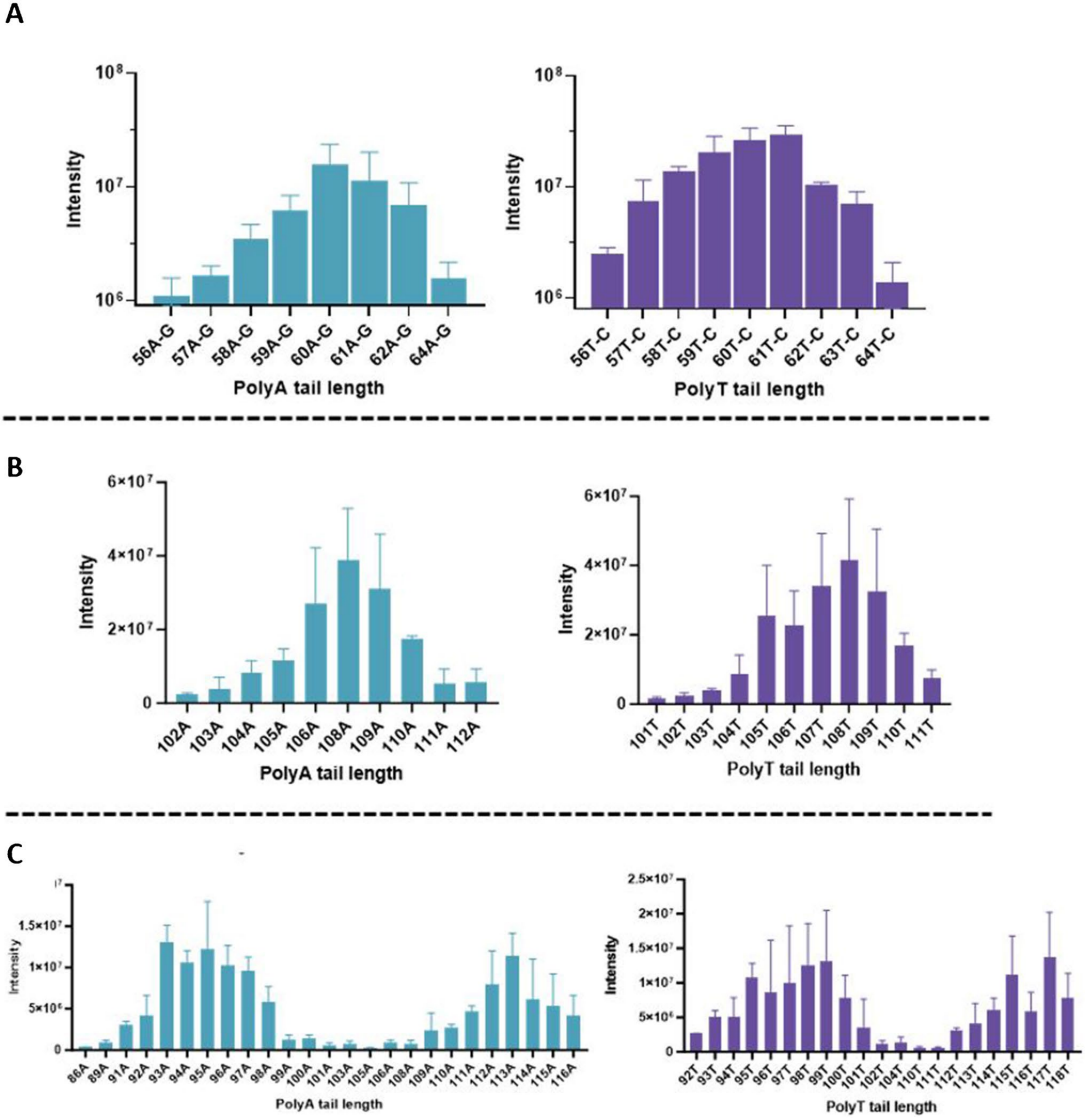
Graphs of average mass spectral signal intensity versus poly(A) and poly(T) tail lengths for three different plasmids with encoded poly(A) of **A** 60A-G, **B** 108A, and **C** 95A. Error bars are shown as the standard deviation from triplicate measurements

The digested double-stranded DNA fragments were next analyzed by IP-RP-LC-HRMS (Fig. 3). Fragment samples of the three plasmids were analyzed in triplicates. Interestingly, as described in Fig. 6, for plasmid with encoded poly(A) of 108A, a total ion current (TIC) chromatogram revealed the presence of several peaks, which corresponded to undigested, partially digested with ClaI, and fully digested plasmids, after reprocessing of the LC-MS data. The same pattern was also observed with the 60A-G and 95A encoding plasmids (data not shown) and confirmed the results obtained by gel electrophoresis. Nevertheless, the interpretation of the LC-MS data from the digested products enabled us to characterize the encoded poly(A) and poly(T) tails for the three plasmids. For example, for the plasmid containing a theorical tail of 108A, the interpretation of a deconvoluted mass spectrum of ClaI/HindIII fully digested DNA products confirmed the 108A tail length (Fig. 6). Interestingly, the LC-MS data also disclosed poly(A) tails of different lengths, both shorter and longer than the expected poly(A) tail length. Indeed, as described in Fig. 7, many deconvoluted masses were observed and separated by the mass of deoxyadenosine (313 ± 2 amu) or deoxythymidine (304 ± 2 amu). Then, poly(A) and poly(T) distributions were also characterized in the two other plasmids with encoded poly(A) 60A-G and 95A (Fig. 8).

Surprisingly, the distribution of poly(A) for the 95A poly(A) DNA template identified two distinct populations around the 95A and 113A tail lengths (Fig. 8C). In fact, the clone containing 95A plasmid was obtained from an initial plasmid preparation containing 112A that lost one adenosine during culture passages in *E. coli*. LC-MS unequivocally revealed the presence of these poly(A) and poly(T) distributions, unlike the other methods used in this study.

## Conclusions

As poly(A) tail is essential for the translation and stability of mRNA, it must be assessed as part of mRNA-based therapeutics and vaccines manufacturing. As of now, Sanger sequencing is the method of choice to analyze the poly(A) tail length in plasmid DNA. However, it does not allow systematic detection of minor poly(A) populations. Because of this, an in vitro LC-MS method was developed and presented in this study as a way to characterize the heterogeneity of poly(A) and poly(T) directly in the plasmid DNA and at a single-nucleotide resolution. In this study, the poly(A) tail lengths of three DNA templates containing poly(A) tails of 60A-G, 95A, and 108A were studied using Sanger sequencing, LC-MS, agarose, acrylamide gels, and capillary electrophoresis. The LC-MS method allowed us to accurately determine the minor and major poly(A) and poly(T) distributions, unlike the electrophoresis-based methods and Sanger sequencing (Supplementary Table 3). Neither approach had the resolution to precisely assess the poly(A) tail lengths and minor poly(A) populations. The analytical strategy employed in this work can be applied to any plasmid type (as shown in this study) or to linear DNA fragments by amplifying the regions of interest using PCR then directly analyzed by HPLC/MS. Poly(A) tail distributions in mRNA had been previously analyzed by HPLC/MS^10^. However, to the best of our knowledge, it is the first time HPLC/MS is used to determine poly(A) tail length directly from plasmid DNA. Another advantage of this method is the observation of a distribution centered on the theoretical number of A, even if the amplification step can introduce a bias. This powerful technique is bound to be useful to characterize the DNA matrix used to perform the IVT in the context of mRNA vaccine development.

## Supplementary Information

Below is the link to the electronic supplementary material.

Supplementary file1 (DOCX 23 KB)
