# Supplementary material for: Characterization of poly(A) and poly(T) tail lengths in plasmid DNA by liquid chromatography high-resolution mass spectrometry
Source: Anal Bioanal Chem. 2024 Nov 16;417(1):59–68. doi: 10.1007/s00216-024-05654-6 (PMC11695563; doi:10.1007/s00216-024-05654-6)
Supplement: Supplementary file 1 — Supplementary file1 (DOCX 23 KB) [file 216_2024_5654_MOESM1_ESM.docx]

**Supplementary Materials**

**Supplementary Table 1**: **Plasmid description.** The description of the principal elements of the plasmids.

bp, base pairs; CDS, coding sequence; IVT, in vitro transcription; ori, origin of replication; RNA pol, RNA polymerase; UTR, untranslated region.

**Supplementary Table 2**: **Size of DNA products after PCR and enzymatic digestion.** The size and description of different species after PCR and digestion.

| **sample Fw/Rev on plasmid** | **ds/ss** | **length (nt)** | **sample Fw/Rev on plasmid** | **ds/ss** | **length (nt)** |
| --- | --- | --- | --- | --- | --- |
| HA(H3)_60AG | | | GFP_ 108A | | |
| Fw+Rev undigested | ds | (249+249) | Fw+Rev undigested | ds | (296+296) |
| Fw+Rev total digestion | ds | (77+79) | Fw+Rev total digestion | ds | (124+126) |
| Fw+Rev dig by Clal | ds | (138+136) | Fw+Rev dig by Clal | ds | (185+183) |
| Fw+Rev dig by Hindlll | ds | (188+192) | Fw+Rev dig by Hindlll | ds | (235+239) |
| Fw undigested | ss | 249 | Fw undigested | ss | 296 |
| Fw total digestion | ss | 77 | Fw total digestion | ss | 124 |
| Fw dig by Clal | ss | 138 | Fw dig by Clal | ss | 185 |
| Fw dig by Hindlll | ss | 188 | Fw dig by Hindlll | ss | 235 |
| Rev undigested | ss | 249 | Rev undigested | ss | 296 |
| Rev total digestion | ss | 79 | Rev total digestion | ss | 126 |
| Rev dig by Clal | ss | 136 | Rev dig by Clal | ss | 183 |
| Rev dig Hindlll | ss | 192 | Rev dig by Hindlll | ss | 239 |
| HA(H3)_95A | | | **Cleaved Region (HA(H3)_60AG/HA(H3)_95A/GFP_108A)** | | |
| Fw+Rev undigested | ds | (283+283) | upstream Fw Clal | ss | 111 |
| Fw+Rev total digestion | ds | (111+113) | upstream Rev Clal | ss | 113 |
| Fw+Rev dig by Clal | ds | (172+170) | downstream Fw Hindlll | ss | 61 |
| Fw+Rev dig by Hindlll | ds | (222+226) | downstream Rev Hindlll | ss | 57 |
| Fw undigested | ss | 283 |  |  |  |
| Fw total digestion | ss | 111 |  | | |
| Fw dig by Clal | ss | 172 |  |  |  |
| Fw dig by Hindlll | ss | 222 |  |  |  |
| Rev undigested | ss | 283 |  |  |  |
| Rev total digestion | ss | 113 |  |  |  |
| Rev dig by Clal | ss | 170 |  |  |  |
| Rev dig by Hindlll | ss | 226 |  |  |  |

dig, digested; ds, double strand; Fw, forward; Rev, reverse; ss, single strand.

**Supplementary Table 3**: **Pros and Cons of each technique mentioned in this communication.**

| Techniques | Pros | Cons | Technical information |
| --- | --- | --- | --- |
| **LC-MS** | Tail length distribution based on both strands (i.e., poly(A) and poly(T) tails)  Accuracy at single-nucleotide resolution  Analysis of multi-population of poly(A) tails | Complex reprocessing of data | Run time: 33 min/sample  Sample preparation time: <3h  Need for interpretation: 30min/sample |
| **Sanger** | Single-nucleotide resolution  Good precision for non-homopolymer sequences | Manual reprocessing of data  Inaccurate for homopolymer sequences | Run time: 1h/sample  Sample preparation time: <3h  Need for interpretation: 10 min/sample |
| **Electrophoresis analysis** | Short run time  Easy to implement | Size accuracy  No information at single nucleotide-resolution | Run time: 1h  Sample preparation: 10 min  Need for interpretation: 5 min/sample |
